# New constraints on exotic spin-dependent interactions with an ensemble-NV-diamond magnetometer

**DOI:** 10.1093/nsr/nwac262

**Published:** 2022-11-17

**Authors:** Hang Liang, Man Jiao, Yue Huang, Pei Yu, Xiangyu Ye, Ya Wang, Yijin Xie, Yi-Fu Cai, Xing Rong, Jiangfeng Du

**Affiliations:** CAS Key Laboratory of Microscale Magnetic Resonance and School of Physical Sciences, University of Science and Technology of China, Hefei 230026, China; CAS Center for Excellence in Quantum Information and Quantum Physics, University of Science and Technology of China, Hefei 230026, China; CAS Key Laboratory of Microscale Magnetic Resonance and School of Physical Sciences, University of Science and Technology of China, Hefei 230026, China; CAS Center for Excellence in Quantum Information and Quantum Physics, University of Science and Technology of China, Hefei 230026, China; CAS Key Laboratory of Microscale Magnetic Resonance and School of Physical Sciences, University of Science and Technology of China, Hefei 230026, China; CAS Center for Excellence in Quantum Information and Quantum Physics, University of Science and Technology of China, Hefei 230026, China; CAS Key Laboratory of Microscale Magnetic Resonance and School of Physical Sciences, University of Science and Technology of China, Hefei 230026, China; CAS Center for Excellence in Quantum Information and Quantum Physics, University of Science and Technology of China, Hefei 230026, China; CAS Key Laboratory of Microscale Magnetic Resonance and School of Physical Sciences, University of Science and Technology of China, Hefei 230026, China; CAS Center for Excellence in Quantum Information and Quantum Physics, University of Science and Technology of China, Hefei 230026, China; CAS Key Laboratory of Microscale Magnetic Resonance and School of Physical Sciences, University of Science and Technology of China, Hefei 230026, China; CAS Center for Excellence in Quantum Information and Quantum Physics, University of Science and Technology of China, Hefei 230026, China; Hefei National Laboratory, University of Science and Technology of China, Hefei 230088, China; CAS Key Laboratory of Microscale Magnetic Resonance and School of Physical Sciences, University of Science and Technology of China, Hefei 230026, China; CAS Center for Excellence in Quantum Information and Quantum Physics, University of Science and Technology of China, Hefei 230026, China; CAS Key Laboratory for Research in Galaxies and Cosmology, Department of Astronomy, University of Science and Technology of China, Hefei 230026, China; School of Astronomy and Space Science, University of Science and Technology of China, Hefei 230026, China; CAS Key Laboratory of Microscale Magnetic Resonance and School of Physical Sciences, University of Science and Technology of China, Hefei 230026, China; CAS Center for Excellence in Quantum Information and Quantum Physics, University of Science and Technology of China, Hefei 230026, China; Hefei National Laboratory, University of Science and Technology of China, Hefei 230088, China; CAS Key Laboratory of Microscale Magnetic Resonance and School of Physical Sciences, University of Science and Technology of China, Hefei 230026, China; CAS Center for Excellence in Quantum Information and Quantum Physics, University of Science and Technology of China, Hefei 230026, China; Hefei National Laboratory, University of Science and Technology of China, Hefei 230088, China

**Keywords:** quantum sensing, exotic interaction, diamond, nitrogen-vacancy center, beyond the standard model

## Abstract

Laboratory search of exotic interactions is crucial for exploring physics beyond the standard model. We report new experimental constraints on two exotic spin-dependent interactions at the micrometer scale based on ensembles of nitrogen-vacancy (NV) centers in diamond. A thin layer of NV electronic spin ensembles is synthesized as the solid-state spin quantum sensor, and a lead sphere is taken as the interacting nucleon source. Our result establishes new bounds for two types of exotic spin interactions at the micrometer scale. For an exotic parity-odd spin- and velocity-dependent interaction, improved bounds are set within the force range from 5 to 500 μm. The upper limit of the corresponding coupling constant $g_A^eg_V^N$ at 330 μm is more than 1000-fold more stringent than the previous constraint. For the *P, T*-violating scalar-pseudoscalar nucleon-electron interaction, improved constraints are established within the force range from 6 to 45 μm. The limit of the corresponding coupling constant $g_S^Ng_P^e$ is improved by more than one order of magnitude at 30 μm. This work demonstrates that a solid-state NV ensemble can be a powerful platform for probing exotic spin-dependent interactions.

## Teaser

A laboratory search of exotic spin-dependent interactions was performed using an ensemble of nitrogen-vacancy centers in diamond, and new constraints at the micrometer scale were established.

## INTRODUCTION

Experimental search of interactions beyond the standard model (SM) has attracted broad interest in recent years [1,2]. Numerous theoretical models indicate long-range interactions beyond the SM that can be mediated by new bosons, such as axions [3–5], familons [6], majorons [7,8] and dark photons [9]. Among the well-motivated theoretically predicted particles, the axion addresses the strong CP violation problem in quantum chromodynamics and is one of the prominent candidates of dark matter [10], as well as a generic prediction of string theories [11,12]. The exchange of hypothetical particles gives rise to exotic spin-dependent interactions between fermions, which were first proposed by Moody and Wilczek [13] and have been analyzed in the form according to polarization and the relative velocity between interacting fermions [14].

The exotic spin-dependent interactions lead to multiple sensor-sensitive effects such as effective magnetic fields sensed by spin systems, and can therefore be investigated via advanced precision measurement experiments in the laboratory [1]. Experimental searches have been conducted using a wide variety of precision measurement technologies, employing the torsion pendulum [15–17], nuclear magnetic resonance [18], trapped ions [19], spin-exchange-relaxation-free atomic magnetometer [20], comagnetometers [21,22] and other high-precision measurement methods [23–26]. These laboratory experiments with broadly developed techniques and devices are pivotal for extending new physics beyond the SM of particle physics and offer an avenue to tests of fundamental physics that is complementary to astronomical searches. Recently, single nitrogen-vacancy (NV) centers in diamond have been developed as single-spin quantum sensors [27] to search for exotic spin-dependent interactions at the micrometer scale [28–30]. The upper bounds were mainly limited by the sensitivity of the sensor. Ensembles of NV centers in diamond have been employed for high-sensitivity measurements of magnetic fields [31–34]. An ensemble-NV-diamond magnetometer provides better sensitivity than a magnetometer using a single NV center [32]. The extension from single NV centers in diamond to an NV ensemble is significant to promote and reinforce the search for exotic spin-dependent interactions. However, the experimental implementation remains elusive.

In this work, we experimentally conducted a search for two types of exotic spin-dependent interactions at the micrometer scale with an ensemble-NV-diamond magnetometer. The exotic interactions between an electron spin and a nucleon can be described as [14,35]


(1) \begin{eqnarray*} V_{AV} = g_A^eg_V^N\frac{\hbar }{4 \pi }\bigg ( \frac{e^{{-{r}/{\lambda }}}}{r}\bigg )\boldsymbol {\sigma }\cdot \boldsymbol {v} , \end{eqnarray*}

(2) \begin{eqnarray*} V_{SP} = g_S^N g_P^e\frac{\hbar ^2}{8 \pi m_e}\bigg (\frac{1}{\lambda r}+\frac{1}{r^2}\bigg ) e^{{-{r}/{\lambda }}} \boldsymbol {\sigma } \cdot \boldsymbol {e_r},\\ \end{eqnarray*}

where $g_A^e$ ($g_V^N$) is the axial-vector (vector) coupling constant of new bosons to electrons (nucleons), and $g_P^e$ ($g_S^N$) is the pseudoscalar (scalar) coupling constant; λ = ℏ/*mc* is the force range with *m* being the mass of the hypothetical particle and *c* the speed of light; ℏ is the reduced Planck’s constant; *m_e_* is the mass of the electron, $\boldsymbol {\sigma }$ is the Pauli vector of the electron spin, $\boldsymbol {v}$ is the relative velocity, **r** is the displacement vector between the electron and nucleon, *r* = |**r**| and $\boldsymbol {e_r}={\bf r}/r$. The exotic parity-odd spin- and velocity-dependent interaction *V_AV_* can be mediated by the exchange of massive spin-1 bosons. The exploration of *V_AV_* is of great importance in providing a new source of parity symmetry violation [1,14], which may help extend the understanding of the mirror universe [36]. The first parity-odd interaction was revealed in the Wu experiment in the weak interaction sector [37]. This interaction vanishes at the micrometer scale. The *P, T*-violating scalar-pseudoscalar interaction *V_SP_* can be mediated by the exchange of spin-0 bosons. The exotic interactions investigated in this work induce effective magnetic fields sensed by the electron spins of the NV centers,


(3) \begin{eqnarray*} {\bf B}_{{\it eff,AV}}({\bf r})= \frac{g_A^eg_V^N}{2 \pi \gamma _e} \bigg ( \frac{e^{{-{r}/{\lambda }}}}{r}\bigg ) \boldsymbol {v} , \end{eqnarray*}

(4) \begin{eqnarray*} {\bf B}_{{\it eff,SP}}({\bf r})= g_S^N g_P^e\frac{\hbar }{4 \pi m_e\gamma _e} \bigg (\frac{1}{\lambda r}+\frac{1}{r^2}\bigg ) e^{{-{r}/{\lambda }}} \boldsymbol {e_r},\\ \end{eqnarray*}

where γ_*e*_ is the gyromagnetic ratio of the electron spins.

## DETECTING THE EFFECTIVE MAGNETIC FIELDS

**Figure 1.**
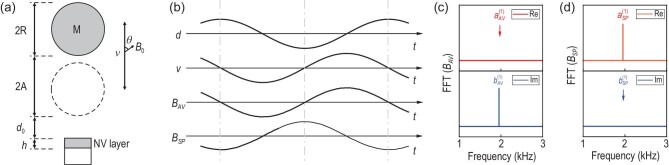
Experimental detection scheme. (a) Schematic experimental parameters. The lead sphere with radius *R* is denoted as M. The vibration amplitude is *A*, and *d*_0_ is the minimal distance between the bottom of M and the surface of the diamond; *h* is the thickness of the layer with doped NV centers; $\boldsymbol {v}$ is the relative velocity vector between the sensor and the lead sphere. A static magnetic field *B*_0_ is applied along the symmetry axis of NV centers. The angle between *B*_0_ and the velocity vector is denoted θ. (b) Time evolutions of the distance *d*(*t*), the velocity *v*(*t*), and the estimated magnetic fields *B_AV_*(*t*) and *B_SP_*(*t*). (c),(d) Fourier transform spectra of *B_AV_*(*t*) and *B_SP_*(*t*), respectively. For *B_AV_*, the non-zero component in the imaginary part corresponds to $b_{{AV}}^{(1)}$. For *B_SP_*, the non-zero component in the real part corresponds to $a_{SP}^{(1)}$.

The detection scheme in our experiment is shown in Fig. 1. The schematic diagram of the nucleon source and NV ensemble is shown in Fig. 1(a). The time-dependent distance between the bottom of the sphere and the surface of the diamond is *d*(*t*) = *d*_0_ + *A*[1 + cos (2π*f_M_t*)], where *d*_0_ is the minimal distance between the bottom of the sphere and the surface of the diamond, and *A* and *f_M_* are the amplitude and frequency of the vibration, respectively. The velocity of the sphere can be represented as *v*(*t*) = −2π*f_M_A*sin (2π*f_M_t*). The possible effective magnetic fields *B_AV_* and *B_SP_* sensed by the sensor can be estimated by integrating (3) and (4) over all nucleons in the sphere and electrons in the NV-doped layer (the detailed data and analysis have been included in the online supplementary material). The time evolutions of the effective magnetic fields *B_AV_*(*t*) and *B_SP_*(*t*) are shown in Fig. 1(b), together with the evolutions of the distance *d*(*t*) and the velocity *v*(*t*). The effective magnetic fields *B_AV_* and *B_SP_* can be decomposed into


(5) \begin{eqnarray*} B_{AV}&=& \sum _n \Big[a_{AV}^{(n)} \cos (2\pi nf_Mt)\\ &&+ b_{AV}^{(n)} \sin (2\pi nf_Mt)\Big], \end{eqnarray*}

(6) \begin{eqnarray*} B_{SP}&=& \sum _n \Big[a_{SP}^{(n)} \cos (2\pi nf_Mt)\\ &&+ b_{SP}^{(n)} \sin (2\pi nf_Mt)\Big], \end{eqnarray*}

where $a_{{AV(SP)}}^{(n)}$ and $b_{{AV(SP)}}^{(n)}$ are Fourier coefficients of the *n*th harmonic. The non-zero amplitudes of Fourier series of *B_AV_* and *B_SP_* mainly lie in the $b_{{AV}}^{(1)}$ and $a_{{SP}}^{(1)}$ components, according to our experimental parameters (see online supplementary material). The component with frequency 1.953 kHz in the imaginary (real) part of the Fourier transform spectrum corresponds to $b_{{AV}}^{(1)}$ ($a_{{SP}}^{(1)}$), as shown in Fig. 1(c) and (d). This feature enables us to search for the signal of the effective magnetic fields *B_AV_* and *B_SP_*.

## RESULTS

**Figure 2.**
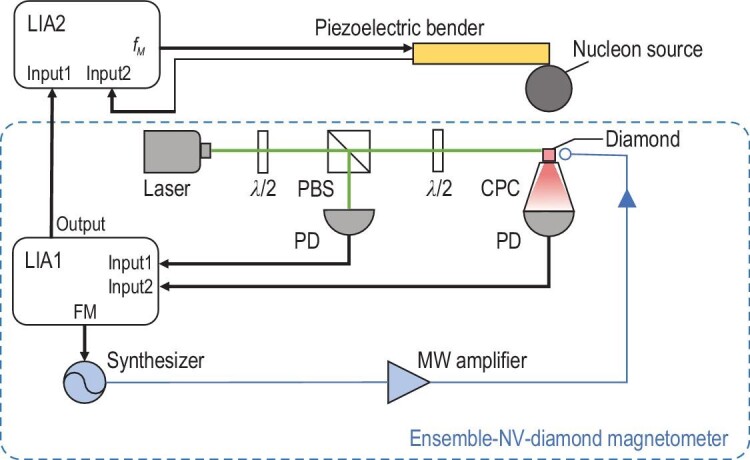
The schematic of our experimental setup. The blue dashed box represents the ensemble-NV-diamond magnetometer. The 532-nm laser illuminates the layer of ensemble NV centers at the diamond surface. The red fluorescence is collected by a compound parabolic concentrator at the bottom. Here λ/2 denotes the half-wave plate, PBS is the polarizing beam splitter, PD is the photodiode, CPC is the compound parabolic concentrator, LIA1(2) is the lock-in amplifier, FM is the modulation frequency of the microwave and *f_M_* is the modulation frequency of the nucleon source.

Our experimental setup based on an ensemble-NV-diamond magnetometer is shown in Fig. 2. A high-purity lead sphere with a radius of *R* = 978(3) μm was taken as the nucleon source with the density of nucleons being 6.8 × 10^30^ m^−3^ [22]. The lead sphere was attached to a piezoelectric bender, which can vibrate at frequency *f_M_* = 1.953 kHz. The sensor is an ensemble of NV centers in a 23-μm-thick layer at the surface of the diamond. The substrate was a high purity electronic grade 〈100〉 oriented single crystal diamond with ppb nitrogen density. A 23-μm-thick nitrogen-rich layer was grown on the surface via the chemical vapor deposition method. After electron irradiation and thermal annealing, a layer of NV ensemble with a concentration of 14 ppm was obtained. The size of the NV layer is 660 × 661 × 23 μm^3^. The number of NV centers that constitute the magnetometer can be estimated to be about 6 × 10^12^. A 532-nm laser with a diameter of 0.8 mm illuminated the NV-doped layer via the flank of the diamond. The red fluorescence from the NV centers was collected by a compound parabolic concentrator [38], filtered by a long-pass filter and detected by a photodetector (PD). A bias magnetic field *B*_0_ of 20 gauss along one of four symmetry axes of NV centers was applied. The microwave field was applied to the NV ensemble via a double split-ring resonator [39]. When the bias magnetic field strength was adjusted to 20 gauss, the fluorescence of the NV ensemble varies as the external magnetic field changes; thus, the NV ensemble can serve as a magnetometer. The possible minor noise component with frequency *f_M_* in *B*_0_ is negligible (a detailed analysis is given in the online supplementary material). In our experiment, to overcome low-frequency noise, the frequency of microwave from the synthesizer was modulated with a frequency of 87.975 kHz. The signal of the PD, which detected the fluorescence from NV centers, was demodulated by the first lock-in amplifier (LIA1) with demodulation frequency 87.975 kHz and time constant 8 μs. Another PD was used to monitor the power fluctuation of the laser for further noise cancelation. The diamond sensor, laser excitation, microwave generation and LIA1 detection system constitute the ensemble-NV-diamond magnetometer. Then the output of the LIA1 was demodulated by the second lock-in amplifier (LIA2) with a reference signal *V_ref_* = *V*_0_cos (2π*f_M_t* + φ)], where φ = 54° ± 9° was the experimentally calibrated phase shift between the output signal of the magnetometer and *d*(*t*). The specific information about the devices utilized in our setup and the calibration of the phase shift are included in the online supplementary material. The time constant of LIA2 was set to be 10 ms. With such a detection method, the in-phase and quadrature components of the demodulated signal from LIA2 corresponds to *B_SP_* and *B_AV_*, respectively.

In our experiment, the sensitivity of the ensemble-NV-diamond magnetometer is 1.4 nT/Hz^1/2^ within the frequency range from 0.4 to 2 kHz. The vibration amplitude was *A* = 718(7) nm, determined by a commercial laser vibrometer. The minimal distance between the bottom of M and the surface of the diamond was *d*_0_ = 9.3(5) μm, set by a vertically installed piezo motor carrying the piezoelectric bender. To estimate the scale of the effective magnetic fields, we take $g_A^eg_V^N = 10^{-20}$, $g_S^Ng_P^e = 10^{-20}$, λ = 10^−4^ m and *f_M_* = 1.953 kHz as an example to calculate the effective magnetic fields with (3) and (4) by numerical integration. The calculated $b_{AV}^{(1)}$ is 9.62 pT, and $a_{SP}^{(1)}$ is 5.24 pT. A more detailed analysis of the calculations of the effective magnetic fields is included in the online supplementary material.

**Figure 3.**
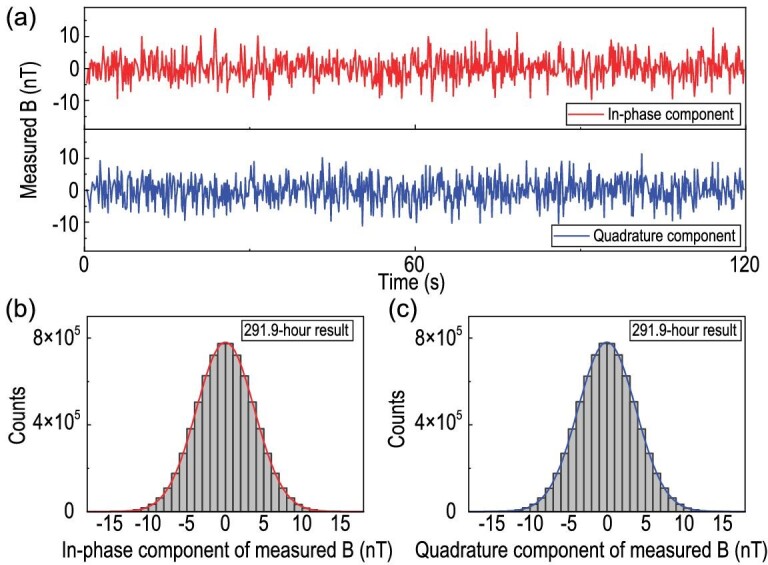
(a) Measurement of the two effective magnetic fields. The red (blue) line corresponds to *B_SP_* (*B_AV_*), which is from the in-phase (quadrature) component of the output of the second lock-in amplifier. (b),(c) Histograms of experimental results for 291.9-h data. The red and blue solid lines are fits to the Gaussian distributions. The averages and the standard errors of the *B_SP_* and *B_AV_* are −1.3 ± 1.4 pT and 0.1 ± 1.4 pT, respectively.

In Fig. 3(a), the in-phase and quadrature parts of the output from LIA2 have been presented with the time duration being 120 s. The total experimental measurement was performed for 291.9 h to reduce statistical uncertainty by averaging fluctuations. The histograms of the experimentally measured effective magnetic fields are shown in Fig. 3(b) and (c). The fits with Gaussian distribution to the histograms for 291.9-h data provide the mean values and the standard errors of the effective magnetic fields. The experimental $B_{SP}^{exp}$ is −1.3 ± 1.4 pT, and the measured $B_{AV}^{exp}$ is 0.1 ± 1.4 pT. For the force range λ = 30 μm, the coupling $g_S^{N}g_P^{e}$ is estimated to be (−7.6 ± 8.2) × 10^−21^, and the coupling constant $g_A^{e}g_V^{N}$ at λ = 330 μm is (0.87 ± 12.25) × 10^−23^. Our results show no evidence of the existence of the exotic spin-dependent interactions, and experimental limits on both interactions can be obtained.

The systematic errors of our experiment are summarized in Table 1, where we take λ = 330 μm for $g_A^{e}g_V^{N}$ and λ = 30 μm for $g_S^{N}g_P^{e}$ as an example. One possible systematic error is due to the diamagnetism of the lead sphere in a bias magnetic field of 20 gauss. The DC component of the magnetic field due to diamagnetism induces an NV ensemble continuous-wave spectrum linewidth broadening. The linewidth broadening is less than 0.4 kHz across the sensing area, which is much smaller than the linewidth in our experiment. The vibration of the lead sphere leads to an AC magnetic field due to diamagnetism. Such an AC magnetic field can only appear in the in-phase component rather than the quadrature component of our measurement, which may affect the result of *B_SP_* rather than that of *B_AV_*. The AC component of the magnetic field due to diamagnetism is less than 0.5 pT, which is smaller than the standard error of the measured field under current statistics. Thus, both the DC component and AC component of the effect of diamagnetism are unobservable in our experiment. The detailed analysis of diamagnetism is included in the online supplementary material. Other systematic errors in our experiment come from the uncertainties of the setup parameters, such as the thickness of the NV layer. To reduce the uncertainties of the thickness, we utilized a thin-layer NV ensemble, whose thickness can be precisely controlled by the parameters during sample growth. We also consider the uncertainties of the angle between the effective magnetic field and the NV axis, the distance between the bottom of M and the surface of the diamond, the radius of M, the vibration amplitude and the misalignment between the center of the diamond and the lead sphere in the *x*-*y* plane. A detailed analysis of the systematic errors is included in the online supplementary material. By combing the systematic errors in quadrature, the total systematic error for $g_A^{e}g_V^{N}$ ($g_S^{N}g_P^{e}$) is derived to be ±4.2 × 10^−25^ (±3.1 × 10^−21^). The bound for the coupling constant $g_A^{e}g_V^{N}$ with λ = 330 μm is $|g_A^{e}g_V^{N}|\le 2.5\times 10^{-22}$ with a 95% confidence level when both statistical and systematic errors are taken into account. The limit for the coupling constant $g_S^{N}g_P^{e}$ with λ = 30 μm is $|g_S^{N}g_P^{e}|\le 2.5\times 10^{-20}$ with a 95% confidence level. Other values of the upper bound with different values of force range can be derived with the same procedure.

**Table 1.** Summary of the systematic errors. The corrections to $g_A^{e}g_V^{N}$ with λ = 330 μm and $g_S^{N}g_P^{e}$ with λ = 30 μm are listed.

		${\Delta g_A^{e}g_V^{N}}$	${\Delta g_S^{N}g_P^{e}}$
Parameter	Value	(×10^−23^)	(×10^−21^)
Diamagnetism	−1.6 × 10^−5^	±0.003	±2.9
θ	54.7° ± 1.3°	${+0.029}$	±0.4
		${-0.028}$	
Distance	9.3 ± 0.5 μm	±0.002	±0.4
Radius	978 ± 3 μm	±0.002	±0.3
Thickness	23 ± 1 μm	±0.002	${+0.3}$
			${-0.4}$
Amplitude	718 ± 7 nm	${+0.008}$	${+0.3}$
		${-0.010}$	${-0.4}$
Deviation	0 ± 10 μm	±0.002	${+0.3}$
			${-0.4}$
Phase delay φ	54° ± 9°	${+0.026}$	±0.3
		${-0.006}$	
Calibration constant	$({2.29\pm 0.03}){\times 10^4 \, \rm V/T}$	±0.012	±0.3
Final $g_A^{e}g_V^{N}\,(\times 10^{-23})$	0.87	$\pm 12.25 \rm \, (stat.)$	
		$\pm 0.04 \rm \, (syst.)$	
Final $g_S^{N}g_P^{e}\, (\times 10^{-21})$	−7.6		$\pm 8.2 \rm \, (stat.)$
			$\pm 3.1 \rm \, (syst.)$

**Figure 4.**
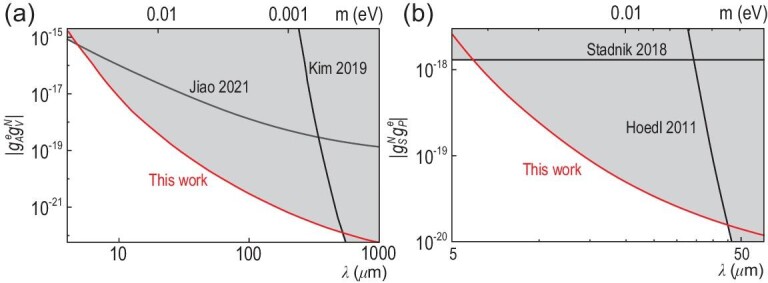
(a) Upper limits on $g_A^eg_V^N$, as a function of the force range λ and mass of the bosons *m*. Black lines are upper limits established by experiments in [20,29]. The red line is the upper bound obtained from our experiment, which establishes an improved laboratory bound in the force range from 5 to 500 μm. (b) Upper limits on $g_S^Ng_P^e$. Black lines are upper limits established by experiments in [16,24]. Our experiment set the most stringent constraints in the force range from 6 to 45 μm.

Figure 4(a) shows the constraints on $g_A^eg_V^N$ set by this work together with limits established by previous experiments. The excluded values of the coupling constant are presented as gray filled areas. The constraints of $g_A^eg_V^N$ for the force range λ < 5 μm were established with a single electron spin sensor by Jiao *et al.* [29]. For force range λ > 500 μm, constraints were set by Kim *et al.* [20], when an optically polarized vapor magnetometer was utilized to detect the possible effective magnetic field from a BGO crystal. For the force range from 5 to 500 μm, the improved experimental limit is established by this work as the red line shown in Fig. 4(a). The upper bound for the force range λ = 330 μm is $|g_A^eg_V^N| \le 2.5 \times 10^{-22}$, which is more than 3 orders of magnitude more stringent than the bound established by the previous result [29]. The major improvement in sensitivity comes from the extension from single NV centers in diamond to an ensemble-NV-diamond magnetometer.

As shown in Fig. 4(b), our work established improved constraints of $g_S^Ng_P^e$ in the force range from 6 to 45 μm. Recent experiments set limits with EDM experiments [24] (λ < 6 μm) and with the torsion pendulum [16] (λ > 45 μm). The upper bound in our experiment at the force range λ = 30 μm is $|g_S^Ng_P^e| \le 2.5 \times 10^{-20}$, which is more than one order of magnitude better than the previous bound established by Stadnik *et al.* [24]. We have noticed that the most stringent constraint on $g_S^Ng_P^e$ may come from the combination of astrophysical observation and laboratory long-range force constraints [40,41]. However, a chameleon mechanism may screen interactions in the space region with high mass density and thus invalidate the astrophysical limits in lab environments [42]. Therefore, it is still crucial to experimentally constrain $g_S^Ng_P^e$ through laboratory measurements [1].

## CONCLUSIONS

In summary, experimental searches for two types of exotic spin-dependent interactions between polarized electrons and unpolarized nucleons have been performed with an ensemble-NV-diamond magnetometer. Improved constraints of two types of coupling constants have been established at the micrometer scale. The current searching sensitivity is mainly limited by the sensitivity of the magnetometer. In the future, the sensitivity of our magnetometer can be improved. Firstly, the concentration of NV centers in diamond can be optimized to enhance the coherence time [32]. Secondly, the double resonance [43] and the hyperfine structure [34] could be used to further improve the sensitivity. Thirdly, other miscellaneous upgrades are helpful for the advancement, such as increasing the optical pumping rate [44], using a silicon carbide heat sink to cool the diamond to increase the signal contrast [45]. To further reduce the systematic errors, some mixed materials may be adopted to tune the magnetic susceptibility closer to zero so as to attenuate the effect of the diamagnetism [46]. Our setup can also be utilized to search for other exotic spin-dependent interactions, such as an exotic parity-even spin- and velocity-dependent interaction between polarized electrons and unpolarized nucleons, and exotic interactions between polarized electrons. Our work shows that an ensemble of NV centers will not only be an important quantum sensor for physics within the standard model, but also be a promising platform for search of new interactions predicted by theories beyond the standard model.

## Supplementary Material

nwac262_Supplemental_File

## SUPPLEMENTARY DATA

Supplementary data are available at *NSR* online, which includes [18,20,45,47,48], contains details of the calculation of the effective magnetic field, the performance of the magnetometer, and statistical and systematic error analysis.
