# Supplementary material for: New constraints on exotic spin-dependent interactions with an ensemble-NV-diamond magnetometer
Source: Natl Sci Rev. 2022 Nov 17;10(7):nwac262. doi: 10.1093/nsr/nwac262 (PMC10232048; doi:10.1093/nsr/nwac262)
Supplement: nwac262_Supplemental_File [file nwac262_supplemental_file.pdf]

Supplemental Data for ‘New Constraints on Exotic Spin-Dependent Interactions with an Ensemble-NV-Diamond Magnetometer’

Hang Liang,[1,2] Man Jiao,[1,2] Yue Huang,[1,2] Pei Yu,[1,2] Xiangyu Ye,[1,2] Ya Wang,[1,2,3] Yijin Xie,[1,2] Yi-Fu Cai,[4,5] Xing Rong,[1,2,3,*] and Jiangfeng Du[1,2,3,†]

[1] *CAS Key Laboratory of Microscale Magnetic Resonance and School of Physical Sciences, University of Science and Technology of China, Hefei 230026, China*

[2] *CAS Center for Excellence in Quantum Information and Quantum Physics, University of Science and Technology of China, Hefei 230026, China*

[3] *Hefei National Laboratory, Hefei 230088, China*

[4] *CAS Key Laboratory for Research in Galaxies and Cosmology, Department of Astronomy, University of Science and Technology of China, Hefei 230026, China*

[5] *School of Astronomy and Space Science, University of Science and Technology of China, Hefei 230026, China*

I. NUMERICAL CALCULATION OF THE EFFECTIVE MAGNETIC FIELDS

In this section, we perform numerical calculation of the possible effective magnetic fields due to the exotic spin-dependent interactions. The effective magnetic fields between electron spin and nucleon are shown in Eq. (3) and (4) in the main text. By Integrating over the volume of both the lead sphere and NV layer, we derive the possible effective magnetic fields $\mathbf{B}_{AV}$ and $\mathbf{B}_{SP}$ sensed by the NV ensemble as follows,

$$\mathbf{B}_{AV} = \frac{1}{V_S}\int_{V_S}\int_{V_M} \mathbf{B}_{eff,AV}(\mathbf{r})\rho_M dV_M dV_S, \quad (1)$$

$$\mathbf{B}_{SP} = \frac{1}{V_S}\int_{V_S}\int_{V_M} \mathbf{B}_{eff,SP}(\mathbf{r})\rho_M dV_M dV_S, \quad (2)$$

where $\rho_M = 6.8\times10^{30}$ m^{-3} is the nucleon density of the lead sphere. V_S and V_M are integral volume of NV layer and lead sphere, respectively. The radius of the lead sphere is $R = 978(3)$ μm. The size of NV layer is $660\times661\times23$ μm^3. The minimal distance d_0 is 9.3(5) μm.

The Monte Carlo method is utilized to numerically calculate the effective magnetic fields to avoid complex calculations due to high integral dimensions [1]. The algorithm of Monte Carlo integral is performed as follows:

(1) $N_{MC} = 2^{20}$ random pairs of points inside both the volumes of the lead sphere and the NV ensemble are generated.

(2) The effective magnetic field $B^i_{eff,\ AV}$ ($B^i_{eff,\ SP}$) between a randomly generated pair of points is calculated with a given force range.

$$B^i_{eff,\ AV} = \frac{g^e_A g^N_V}{2\pi\gamma_e}\frac{e^{-\frac{r}{\lambda}}}{r} v cos\theta, \quad (3)$$

$$B^i_{eff,\ SP} = g^N_S g^e_P \frac{\hbar}{4\pi m_e\gamma_e}\left(\frac{1}{\lambda r}+\frac{1}{r^2}\right)e^{-\frac{r}{\lambda}}\frac{z}{r}cos\theta, \quad (4)$$

where $\theta = arccos(1/\sqrt{3})$ is the angle between the direction of the velocity $\mathbf{v}$ and the NV axis.

(3) All the contributions to the effective magnetic fields are summed and normalized to give the average magnetic fields generated by the lead sphere and sensed by the NV ensemble:

$$B_{AV} = N_{nucleon}\frac{1}{N_{MC}}\sum_i^{N_{MC}} B^i_{eff,\ AV}, \quad (5)$$

$$B_{SP} = N_{nucleon}\frac{1}{N_{MC}}\sum_i^{N_{MC}} B^i_{eff,\ SP}, \quad (6)$$

where $N_{nucleon}$ is the total number of nucleons in lead sphere.

The magnetic field B_{AV} and B_{SP} can be decomposed into orthogonal components of Fourier series as shown in Eq. (5) and (6) in the main text, the coefficients can be derived as

$$a^{(n)}_{AV(SP)} = \frac{2}{T}\int_0^T \cos(2\pi n f_M t) B_{AV(SP)}(t)dt, \quad (7)$$

$$b^{(n)}_{AV(SP)} = \frac{2}{T}\int_0^T \sin(2\pi n f_M t) B_{AV(SP)}(t)dt, \quad (8)$$

where $T = 1/f_M$ is the period of the effective magnetic field. We take $g^e_A g^N_V = 1\times10^{-20}$, and $\lambda = 10^{-4}$ m as an example, the coefficients of B_{AV} at first three harmonic frequencies are listed as follows

n	1	2	3
$a^{(n)}_{AV}$ (pT)	0	0	0
$b^{(n)}_{AV}$ (pT)	9.62	0.02	0.00

The amplitude of the first harmonic coefficient $b^{(1)}_{AV}$ is much larger than higher order harmonic coefficients $b^{(2)}_{AV}$ and $b^{(3)}_{AV}$. Values of $a^{(n)}_{AV}$ are zero. Similarly, the calculated coefficients of B_{SP} at the first three harmonic frequencies are

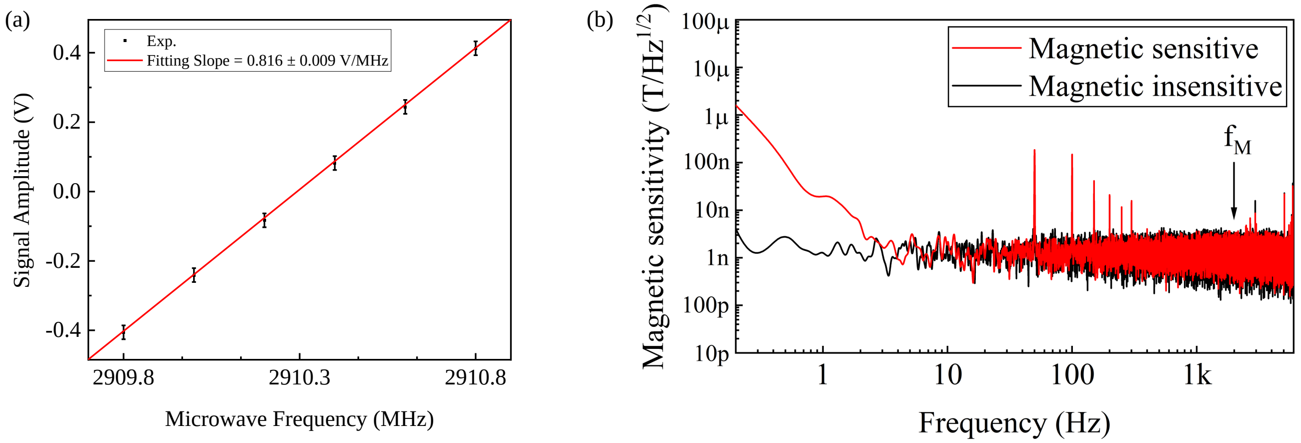

FIG. S1. **The performance of the magnetometer.** (a) The specific region of the CW spectrum. The red line is the linear fitting to give the max slope. (b) Magnetic sensitivity of ensemble-NV-diamond magnetometer. The peaks of the red line at 50 Hz and harmonics are the magnetic noise due to the power supply of the equipment. The vibration frequency of the lead sphere f_M = 1.953 kHz is also displayed.

n	1	2	3
$a_{SP}^{(n)}$ (pT)	5.24	-0.06	-0.06
$b_{SP}^{(n)}$ (pT)	0	0	0

when $g_S^N g_P^e = 1\times10^{-20}$, and $\lambda = 10^{-4}$ m. The amplitude of the first harmonic coefficient $a_{SP}^{(1)}$ is much larger than higher order harmonic coefficients $a_{SP}^{(2)}$ and $a_{SP}^{(3)}$, and values of $b_{SP}^{(n)}$ are zero. The possible effective magnetic fields mainly lie in the first order harmonic coefficients with the experimental parameters in our setup.

II. THE PERFORMANCE OF THE ENSEMBLE-NV-DIAMOND MAGNETOMETER

The ensemble-NV-diamond magnetometer used in our experiment was based on the continuous-wave (CW) method, wherein laser and microwave fields are continuously applied to NV centers. The NV centers of $|m_s = 0\rangle$ state can be transmitted to $|m_s = +1\rangle$ by a resonance microwave with an angular frequency $\omega_e = D + \gamma_e B_0$, where $D = 2\pi \times 2.87$ GHz is the ground-state zero splitting, $\gamma_e = 2\pi \times 28$ GHz/T is the gyromagnetic ratio of the electron spin, and B_0 is the bias magnetic field along the symmetry axis of NV centers generated by a solenoid coil. When the external magnetic field varies, the population on $|m_s = +1\rangle$ states decreases, resulting in changes in fluorescence which can be detected.

In order to avoid flicker noise, the frequency modulation technique was used in our experiment. The frequency of microwave from the synthesizer was modulated by a lock-in amplifier (LIA1 in Fig. 2 of the main text) with modulation frequency FM being 87.975 kHz. The signal of PD, which detected the fluorescence from NV centers, was demodulated with the same frequency. For further noise cancellation, the signal of a reference PD used to monitor the power fluctuation of the laser was also demodulated by LIA1 with a frequency of 87.975 kHz. The laser intensity noise was canceled by scaling and subtracting the demodulated reference signal from the demodulated fluorescence signal, with a cancellation coefficient of about 2 [3].

The calibrated constant of the magnetic field to the output of the magnetometer was determined by the max slope of the CW spectrum, which was 0.816 ± 0.009 V/MHz as shown in Supplementary Fig. S1(a), and corresponds to a calibrated constant of (2.29 ± 0.03) × 10^4 V/T with the gyromagnetic ratio $\gamma_e = 2\pi \times 28$ GHz/T. The sensitivity of 1.4 nT/Hz$^{1/2}$ from 0.4 to 2 kHz was achieved, as shown in Supplementary Fig. S1(b).

Our experiment measured the variation in amplitude of the external magnetic field at f_M = 1.953 kHz under a static bias magnetic field B_0. As shown in Supplementary Fig. S1(b), the frequency component with frequency f_M in B_0 is clean. Furthermore, our measurement was in phase with the vibration of the lead sphere. The variation of B_0 was asynchronous with the vibration. During 291.9-hour experimental measurement, the frequency component at 1.953 kHz in B_0 did not occur due to uncorrelated random phase. Besides, the possible minor noise of B_0 at f_M was included in the measurement result together with the target effective magnetic fields. Our final zero result also showed no effect of the variation of B_0 at f_M. In conclusion, the possible minor noise of B_0 at 1.953 kHz is negligible in our experiment.

Instrument	Manufacturer	Model
Lock-in Amplifier1 and 2	Zurich Instruments	HF2LI
Laser	Cobolt	0532-05-01-1500-700
Synthesizer	National Instruments	FSW-0010
MW Amplifier	CIQTEK	GYPA2530-42
PD	Thorlabs	SM05PD1A
Piezoelectric Bender	Harbin Core Tomorrow	NAC2223

TABLE I. Detailed information about the devices in our experimental setup.

III. CALIBRATION OF THE PHASE DELAY

The phase delay ϕ between the output signal of the ensemble-NV-diamond magnetometer and $d(t)$ can be calibrated by a given signal with a method similar to that described in Ref. [2]. The calibration procedure was carried out before the experiments with the lead sphere. A thin copper wire carrying a DC current was stuck to the front section of the piezoelectric bender. The magnetic field generated by the current-carrying copper wire was modulated by the vibration of the piezoelectric bender and thus in phase with $d(t)$. The output signal of the magnetometer and the feedback of the piezoelectric bender, which was used to monitor $d(t)$, were demodulated by a lock-in amplifier (LIA2 in Fig. 2 of the main text) with the same frequency $f_M = 1.953$ kHz. The demodulation signal from LIA2 provided the amplitude of the magneic field as (18 ± 2) nT. The phase delay between the demodulation signal of the output signal of magnetometer and feedback of piezoelectric bender was $\phi_1 = -32(9)°$.

We also use a commercial laser vibrometer (Sunny Optical, LV-S01) to measure $d(t)$. The phase delay between $d(t)$ and feedback of piezoelectric bender was calibrated to be $\phi_2 = -86(1)°$. The phase delay ϕ between the output signal of the magnetometer and $d(t)$ can be obtained to be $\phi = \phi_1 - \phi_2 = 54(9)°$. For experimental operation, considering $d(t)$ was monitored by the feedback of piezoelectric bender, the lock-in amplifier (LIA2) was set with a phase difference $\Delta\phi = \phi_1 = -32°$ between the demodulation phase of the output signal of magnetometer and feedback of piezoelectric bender.

IV. LIST OF EXPERIMENT INSTRUMENTS

The schematic of the experimental setup is shown in Fig. 2 in the main text. Table I shows the manufacturers and models of devices used in our experiment.

V. SYSTEMATIC ERROR ANALYSIS

Diamagnetism of the lead sphere

The diamagnetism of the vibrating lead sphere leads to the modulation of a static magnetic field sensed by the NV ensemble. Both DC component and AC component of the effect of diamagnetism are unobservable in our experiment according to following detailed analyses.

We first calculate the static magnetic field due to diamagnetism among the sensing area of the NV ensemble. With a bias magnetic field of $B_0 = 20$ Gauss, the diamagnetism of the lead sphere causes a magnetic field B_{diam} on each NV center in the layer,

$$\mathbf{B}_{diam} = \int_{V_M} \frac{\chi}{4\pi} \left[\frac{3\mathbf{r}\left(\mathbf{B}_0 \cdot \mathbf{r}\right)}{r^5} - \frac{\mathbf{B}_0}{r^3} \right] dV_M, \qquad (9)$$

where $\chi = -16 \times 10^{-6}$, is magnetic susceptibility of the lead sphere [4], V_M is the integral volume of lead sphere. Since the large zero filed splitting of NV centers, the magnetic field perpendicular to NV axis can be ignored. The magnetic field parallel to NV axis due to diamagnetism is denoted as $B_{diam,\parallel}$. The distribution of $B_{diam,\parallel}$ in NV ensemble is shown in Supplementary Fig. S2.

The DC component of the effect of the diamagnetism leads to an inhomogeneous static magnetic field among the NV ensemble layer. The maximum variation of the magnetic field is 14 nT. Since the measurement of the effective magnetic field shown in Fig. 3 in the main text is obtained after demodulation at the specific frequency $f_M = 1.953$ kHz, the DC component of the magnetic field is filtered out. The static magnetic field due to diamagnetism may induce an NV ensemble CW spectrum linewidth broadening of less than 0.4 kHz across the sensing area, which is much smaller than the megahertz linewidth in our experiment. Therefore, the DC component of the effect of diamagnetism is negligible in our experiment.

Then we calculate the AC component of the magnetic field caused by the diamagnetism of the vibrating lead sphere. The averaged magnetic field sensed by the NV ensemble due to diamagnetism is calculated to be in the range from 0.738 pT to 0.740 pT during the vibration of the lead sphere, corresponding to an AC magnetic field with the amplitude being 0.001 pT, which is much less than the standard error of the measured field under current statistics.

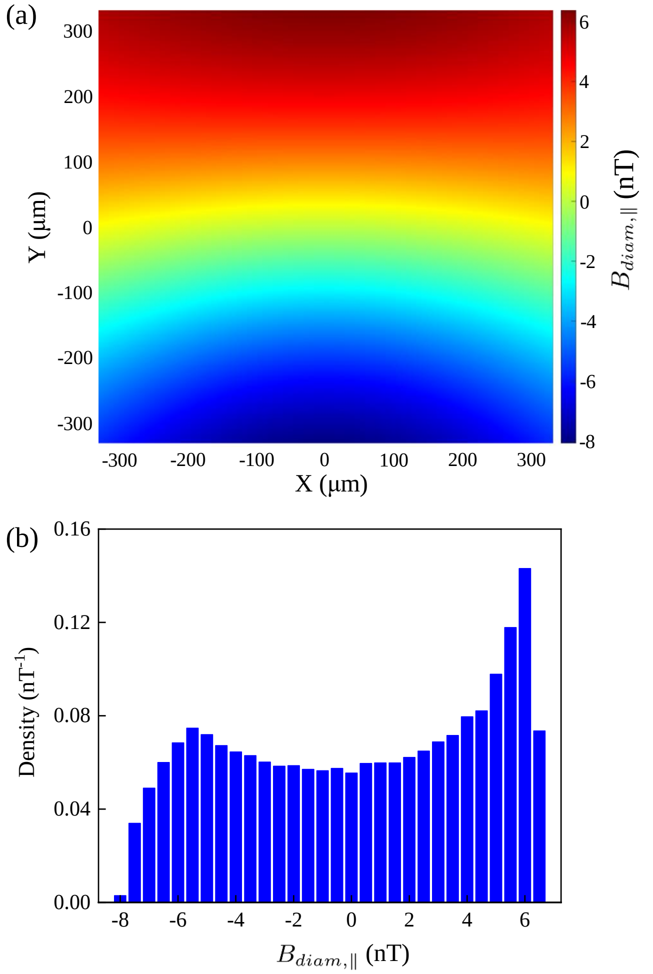

FIG. S2. **The distribution of $B_{diam,\parallel}$ on NV ensemble.** (a) Contour plot of $B_{diam,\parallel}$ in x-y plane. The x-y plane is perpendicular to the vibration of the piezoelectric bender, x-axis stands for the direction perpendicular to the NV axis. (b) The histogram of $B_{diam,\parallel}$.

We also take possible misalignment of the lead sphere and diamond into consideration. The maximum misalignment is estimated to be 10 μm. The linewidth broadening caused by the static magnetic field due to diamagnetism is less than 0.4 kHz and is negligible in our experiment. The magnetic field sensed by the NV ensemble due to diamagnetism is calculated to be in the range from 200.4 pT to 201.4 pT during the vibration of the lead sphere, corresponding to an AC magnetic field with the amplitude being 0.5 pT, which is less than the standard error of the measured field under current statistics.

In conclusion, both DC component and AC component of the effect of diamagnetism are unobservable in our experiment. The magnetic field due to diamagnetism is in phase with $d(t)$ and could only appear in the in-phase component rather than the quadrature component of our measurement. This may affect the result of B_{SP} rather than that of B_{AV}. The correction to $g_S^N g_P^e$ is $(0.0\pm2.9)\times 10^{-21}$ at $\lambda = 30$ μm. Taking uncertainty in phase delay ϕ into account, the correction to $g_A^e g_V^N$ is $(0.0\pm0.3)\times10^{-25}$ at $\lambda = 330$ μm.

Uncertainty in d_0

The distance between the bottom of the lead sphere and the diamond is adjusted by a vertically installed piezo motor (Physik Instrumente, Q-545). The lead sphere first slowly approaches the diamond surface with a tiny vibration amplitude, and the position of slight contact can be detected when the feedback of piezoelectric bender suddenly varies [5]. The lead sphere was then lifted by 10 μm, according to the integrated position sensor of the piezo motor. The minimal distance between the bottom of the lead sphere and the diamond d_0 is 9.3(5) μm since the vibration amplitude is $A =$ 718(7) nm. The uncertainty is due to long time drift of our system.

To estimate the corrections to $g_A^e g_V^N$ and $g_S^N g_P^e$ due to the uncertainty in d_0, 10^5 samples for d_0 was randomly taken, which satisfied a Gaussian distribution $P_{d_0}(d_{0,i}) = \frac{1}{\sqrt{2\pi}\sigma_{d_0}} exp[-\frac{(d_{0,i}-\mu_{d_0})^2}{2{\sigma_{d_0}}^2}]$. $\mu_{d_0} = 9.3$ μm and $\sigma_{d_0} = 0.5$ μm are the mean value and uncertainty of measured d_0. Then the mean values and the standard deviations of $g_A^e g_V^N$ and $g_S^N g_P^e$ can be obtained. The correction to $g_A^e g_V^N$ is $(0.0 \pm 0.2) \times 10^{-25}$ at $\lambda = 330$ μm. The correction to $g_S^N g_P^e$ is $(0.0 \pm 0.4) \times 10^{-21}$ at $\lambda = 30$ μm.

Uncertainty in R

The radius of the lead sphere is measured to be $R = 978(3)$ μm. The correction to $g_A^e g_V^N$ is obtained in the same procedure as that of uncertainty in d_0. The correction to $g_A^e g_V^N$ is $(0.0\pm0.2) \times 10^{-25}$ at $\lambda = 330$ μm. The correction to $g_S^N g_P^e$ is $(0.0\pm0.3) \times 10^{-21}$ at $\lambda =$ 30 μm.

Uncertainty in θ

The angle between the effective magnetic field and the NV axis is 54.7 ± 1.3 °, containing crystallographic orientation of the diamond and the angle between the surface of the diamond and the piezoelectric bender. The uncertainty of θ mainly comes from the tilt of the piezoelectric bender, which can be obtained through our optical system. The correction to $g_A^e g_V^N$ is from -2.8×10^{-25} to 2.9×10^{-25} at $\lambda = 330$ μm. The correction to $g_S^N g_P^e$ is $(0.0\pm0.4) \times 10^{-21}$ at $\lambda = 30$ μm.

Uncertainty in h

The thickness of the NV layer is estimated by measuring the thickness of the diamond before and after the growth of the NV layer. The original thickness of the diamond is 551(1) μm. After the growth of the NV layer, the thickness of the diamond is 574(1) μm. The thickness of the NV layer is measured to be $h = 23(1)$ μm. The correction to $g_A^e g_V^N$ is $(0.0\pm0.2) \times 10^{-25}$ at $\lambda = 330$ μm. The correction to $g_S^N g_P^e$ is from -0.4×10^{-21} to 0.3×10^{-21} at $\lambda = 30$ μm.

Uncertainty in A

The vibration amplitude is measured to be $A = 718(7)$ nm, using a commercial laser vibrometer (Sunny Optical, LV-S01). The correction to $g_A^e g_V^N$ is from -1.0×10^{-25} to 0.8×10^{-25} at $\lambda = 330\ \mu$m. The correction to $g_S^N g_P^e$ is -0.4×10^{-21} to 0.3×10^{-21} at $\lambda = 30\ \mu$m.

Deviation in x-y plane.

The deviation in x-y plane is measured to be $(0\pm10)\ \mu$m according to the CCD images as shown in Fig. S3. The correction to $g_A^e g_V^N$ is $(0.0\pm0.2)\times10^{-25}$ at $\lambda = 330\ \mu$m. The correction to $g_S^N g_P^e$ is -0.4×10^{-21} to 0.3×10^{-21} at $\lambda = 30\ \mu$m.

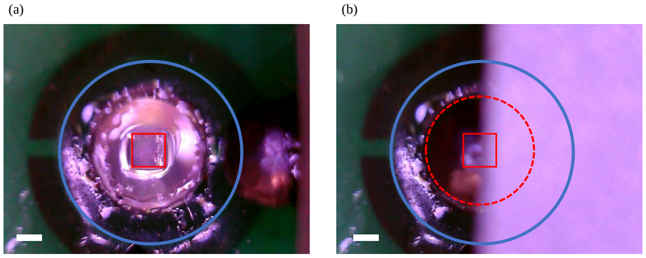

FIG. S3. **CCD image for estimation of the misalignment between the diamond and the lead ball.** (a) CCD image when the lead ball was moved away from above the center of the diamond. The pattern of double-splitring-resonator (the blue line in the figure), which was used to deliver the microwave field in our experiment, is used to mark the relative position of the diamond. The red lines show the location of the diamond. The lead ball is displayed in the right part of the figure. (b) CCD image when the lead ball was located above the center of the diamond. The red dashed line shows the position of the lead ball. The length of the scale bar corresponds to 500 μm.

Uncertainty in ϕ

The phase delay is measured to be $\phi = (54\pm9)$ degree. The correction to $g_A^e g_V^N$ is from -0.6×10^{-25} to 2.6×10^{-25} at $\lambda = 330\ \mu$m. The correction to $g_S^N g_P^e$ is $(0.0\pm0.3)\times10^{-21}$ at $\lambda = 30\ \mu$m.

Uncertainty in Calib. Const.

The calibrated constant of the magnetic field to the output of magnetometer is measured to be $(2.29\pm0.03)\times10^4$ V/T. The correction to $g_A^e g_V^N$ is $(0.0\pm1.2)\times10^{-25}$ at $\lambda = 330\ \mu$m. The correction to $g_S^N g_P^e$ is $(0.0\pm0.3)\times10^{-21}$ at $\lambda = 30\ \mu$m.

With the fiducial probability of 95%, the upper bound $|g_A^e g_V^N| \leq 2.5\times10^{-22}$ for the force range $\lambda = 330\ \mu$m and the upper bound $|g_S^N g_P^e| \leq 2.5\times10^{-20}$ for the force range $\lambda = 30\ \mu$m were obtained, taking both statistical and systematic errors into account.

* xrong@ustc.edu.cn
† djf@ustc.edu.cn

[1] Kim YJ, Chu PH and Savukov I *et al.* Experimental limit on an exotic parity-odd spin- and velocity-dependent interaction using an optically polarized vapor. *Nat Commun* 2019; **10**: 2245.

[2] Su H, Wang T and Jiang M *et al.* Search for exotic spin-dependent interactions with a spin-based amplifier. *Sci Adv* 2021; **7**: eabi9535.

[3] Schloss JM, Barry JF, Turner MJ *et al.* Simultaneous Broadband Vector Magnetometry Using Solid-State Spins. *Phys Rev Applied* 2018; **10**: 034044.

[4] Ruble J. CRC handbook of chemistry and physics. 102nd edition 2021–2022 2021.

[5] Rakotondrabe M, Ivan IA and Khadraoui S *et al.* Simultaneous Displacement/Force Self-Sensing in Piezoelectric Actuators and Applications to Robust Control. *IEEE/ASME Trans Mechatron* 2014; **20**: 519-531.
